# Absorbent Cotton to Facilitate Evacuation of Swallowed Coins

**Published:** 1912-10

**Authors:** 


Absorbent Cotton to Facilitate Evacuation of Swallowed Coins. D'Halluin, *Trib. Med.*, Sept., 1911. A boy of five years had swallowed two five-centime pieces (about the same size as Canadian cents) which were seen by radioscopy in the gastric region. Confections were made with absorbent cotton powdered with bismuth and these the child, after a few hours fasting, swallowed greedily. The shadow thus produced obscured that of the coins, proving that the latter were really in the stomach. Eighteen hours later, a voluminous mass was indicated in the rectum but a smaller mass made a shadow, containing the darker shadows of the coins, in the cæcum. On the second day after administration of the cotton, the coins were passed simultaneously and without a bit of fæcal matter, according to the mother's statement.

